# Salicylic Acid-Induced Expression Profiles of LRR and LRR-RLK Candidate Genes Modulate *Mungbean Yellow Mosaic India Virus* Resistance in Blackgram and Its Two Wild Non-Progenitors

**DOI:** 10.3390/plants13243601

**Published:** 2024-12-23

**Authors:** Mansi Shukla, Priyanka Kaundal, Shalini Purwar, Mukul Kumar, Chandragupt Maurya, Chirag, Awdhesh Kumar Mishra, Kwang-Hyun Baek, Chandra Mohan Singh

**Affiliations:** 1 Department of Genetics and Plant Breeding, Banda University of Agriculture and Technology, Banda 210 001, India; mansishukla034@gmail.com (M.S.); kaundal.priyanka10@gmail.com (P.K.); mukulbreeder@rediffmail.com (M.K.); mauryachandragupt133@gmail.com (C.M.); chirag8054@gmail.com (C.); 2 Department of Basic and Social Sciences, Banda University of Agriculture and Technology, Banda 210 001, India; purwarshalini@gmail.com; 3 Department of Biotechnology, Yeungnam University, Gyeongsan 38541, Republic of Korea

**Keywords:** *YMD*, salicylic acid, gene expression, LRR-RLK, qRT-PCR, R-genes

## Abstract

Blackgram is an important short-duration grain legume, but its yield is highly affected by various stresses. Among biotic stresses, yellow mosaic disease (YMD) is known as a devastating disease that leads to 100% yield loss under severe conditions. The cultivated lines possess resistance, but exploring more diverse sources of resistance may be useful for pyramiding to improve the durability of said resistance. Some wild *Vigna* species have potentially demonstrated a high level of resistance. R-genes, including gene families of leucine-rich repeats (LRRs) and leucine-rich repeat receptor-like kinases (LRR-RLKs), are known for modulating the resistance in plants against various biotic stresses. The first comprehensive analysis of the LRR and LRR-RLK gene families in mungbean is reported in the present study. A total of forty-six candidate genes were identified and grouped into eight clades. Protein motif analysis showed that the “Pkinase domain” and “LRR domains” were conserved in most of the R-proteins. The expression of candidate genes viz. *VrNBS_TNLRR-8*, *VrLRR_RLK-20*, *VrLRR_RLK-17*, and *VrLRR_RLK-19* demonstrated significantly up-regulated expression upon YMD infection in control and salicylic acid-primed (SA-primed) plants. The analysis provides insight into the diversity and robust candidate genes for functional studies modulating YMD resistance altered by salicylic acid.

## 1. Introduction

Blackgram (*Vigna mungo* L. Hepper) is an important pulse crop that matures in a short period and loves humidity. India is the world’s primary producer and consumer of blackgram [1]. Nutritionally, this crop plays an important role in the human diet. The composition of proteins, lipids, starch, and total soluble sugars in blackgram is approximately 43.5, 4.84, 22.0, and 1.1%, respectively. The presence of *Rhizobium* in the roots of urdbean augments the importance of this crop by improving soil health through the fixation of nitrogen [2]. Thus, the economic importance of blackgram cannot be ignored. Considering the importance of this crop, various biotic and abiotic stresses act as hindrances in accomplishing its potential yield. Among the various biotic stresses, YMD is a prime concern that leads to yield loss of 100% under severe conditions [3]. The correct identification of pathogens that lead to a particular disease is a very important step in planning effective mitigation strategies. The causal virus that leads to the development of YMD at our location has already been identified as *MYMIV* in a previous study [4]. To overcome obstacles of classical plant breeding for disease resistance, a trend of induced resistance is currently underway. It includes systemic acquired resistance (SAR) and induced systemic resistance (ISR). Secondary resistance due to SAR is induced after the plant has been exposed to elicitors or chemical stimuli [5]. The reports of previous workers proved that exogenous application of SA can reduce viral symptoms [6]. Hence, salicylic acid can be used efficiently for inducing systemic acquired resistance (SAR) in plants by signal transduction [7]. SA raises the amount of phenolic chemicals, which can impede and strengthen resistance against pathogens [8]. Additionally, it improves the antimicrobial activity of pathogen-related proteins, enhancing resistance to viral infections [9], and also has an impact on physiological processes like photosynthesis [10,11,12], the permeability of membranes, and antioxidant enzyme activity [13]. It could be possible through alterations in responses of candidate genes by the elicitors modulating disease resistance. R-genes have been reported and are well characterized in many crops [14]. R-proteins are formed of five different classes of proteins [15], among which the largest group contains nucleotide binding sites (NBSs), LRRs, or both [16]. The activity of LRRs is increased when the efficiency of these genes receives external stress signals. This is achieved when they are bound to receptors such as RLKs. LRR-RLKs form the largest class of receptors which are actively involved in signal transduction and other processes directly associated with the development of plants [17]. A single LRR-RLK protein contains an extracellular domain (ED), involved in ligand binding, a single-membrane spanning helix (TM), and a cytoplasmic domain (KD) [17]. A typical structure of LRRs contains 23–40 residues of amino acids rich in mostly leucine [18]. All of these amino acid residues form tandem repeats. It also contains an α/β loop that helps in the establishment of protein–protein interaction [19]. LRR-RLK proteins, when bound to specific receptor molecules, lead to the downstream activation of various other kinds of proteins like mitogen-activated protein kinases (MAPKs), WRKY, etc., which participate in the induction of resistance in plants against various biotic factors. LRR-RLKs have been recognized and characterized in many crops like tomato [20], *Arabidopsis* [21], *Glycine max* [22], and two *Citrus* spp. [23], among others. Expression of LRR-RLK genes in various crops is proven to be efficient in enhancing resistance against various diseases. In blackgram, this family is still unexplored due to the non-availability of chromosome-level assembly. In the present study, we utilized the database of its close relative *Vigna radiata* to retrieve the data, and its gene expression was performed in blackgram and its two highly resistant wild relatives. The SA-mediated cross-specific responses of LRRs and LRR-RLKs will provide a new scope for gaining insights into the molecular basis of resistance.

## 2. Results

### 2.1. Genome-Wide Identification and Characterization of R-Genes

R-genes (LRRs and LRR-RLKs) were explored in the mungbean genome (a close ancestor of urdbean). A total of 46 genes were identified which possessed LRR and LRR-RLK domains of interest. Out of 46 candidates, 13 genes had LRRs, whereas 33 genes contained LRR-RLK domains. These 13 NBS-LRR genes could be classified as coiled-coil-domain-containing (*CNL*), Toll/interleukin-1 receptor-like-domain-containing (*TNL*), R-NBS-LRR (*RNL*), *NLRRtir*, *NLRRcc*, and nucleotide-binding site leucine-rich repeat receptor (*NLR*). The characteristic features like gene id, gene start and end points, genomic length (bp), CDS length (bp), linkage group (Ch.), protein length, molecular weight of protein (kD), isoelectric point (pI), and exon number are described in Table 1.

It was noticed that out of 46 genes, 3 genes were present in the scaffold, so they were not mapped on the chromosomes. The maximum genomic length of 18,648 bp was observed for the gene *VrNBS_CNLRR-4,* whereas the minimum length of the genomic region was observed in *VrLRR_RLK-45*, which had a genomic length of only 225 bp. In the case of CDS length, the maximum length of 6866 bp was recorded for *VrLRR_RLK-23*, and the minimum length of 180 bp was recorded for *VrLRR_RLK-28*. Genes *VrLRR_RLK-35* and *VrLRR_RLK-24* had the maximum isoelectric point of 9.97 pH. Protein length and molecular weight were maximum in *VrNBS_CNLRR-4,* which were 2420 AA and 272.73 Da, respectively. *VrNBS_NLRRcc-5* had a maximum of 23 exons, whereas the rest of the genes containing LRR-RLK domains had only 1 exon.

### 2.2. Physical Mapping of R-Genes on Vigna Genome Assembly

**Figure 1:**
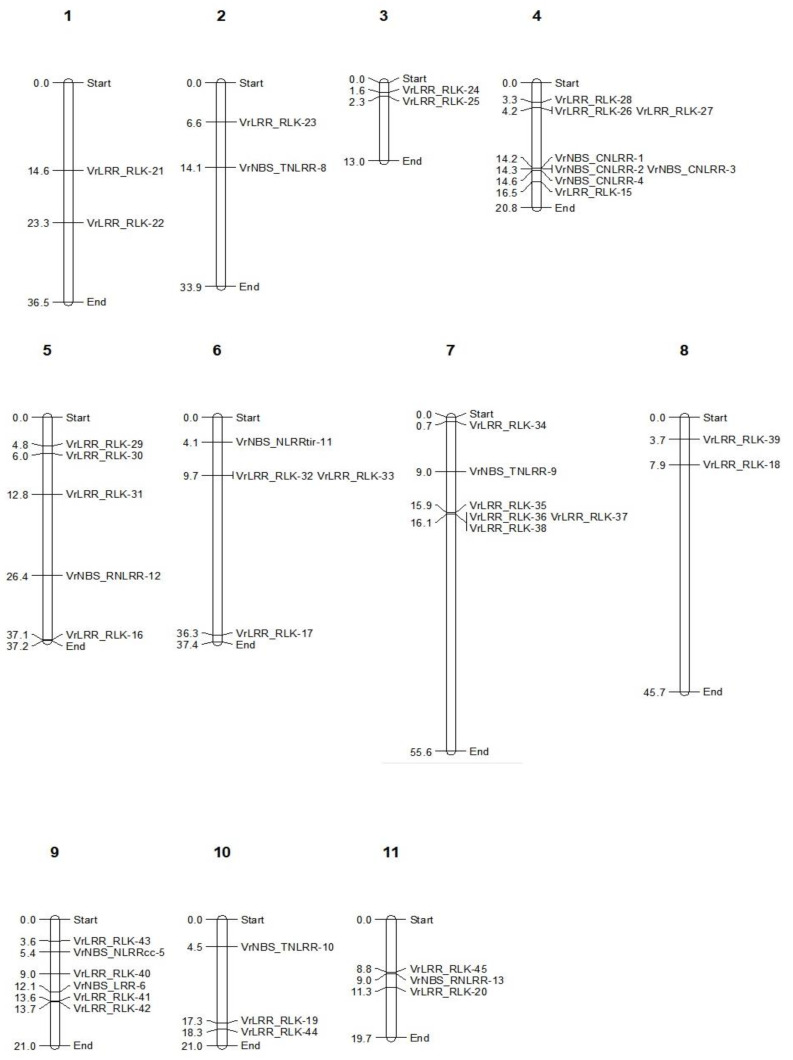
Physical mapping of LRR and LRR-RLK genes on different chromosomes.

The location of total 43 genes could be delineated on 11 different chromosomes of *Vigna radiata*, whereas the rest of the genes could not be mapped due to their presence in the scaffold (Figure 1).

It was found that the maximum number of genes (seven) was present on Ch.4, i.e., three NBS-CNLRR and four LRR-RLK genes, whereas Ch.2 consisted of only one R-gene (VrNBS_TNLRR-8). The LRR-RLK genes were unevenly distributed across all 11 chromosomes. However, NBS-TNLRR genes were mapped on Ch.2 and Ch.7.

### 2.3. Phylogenetic Relationships Among Identified Candidate R-Genes

**Figure 2:**
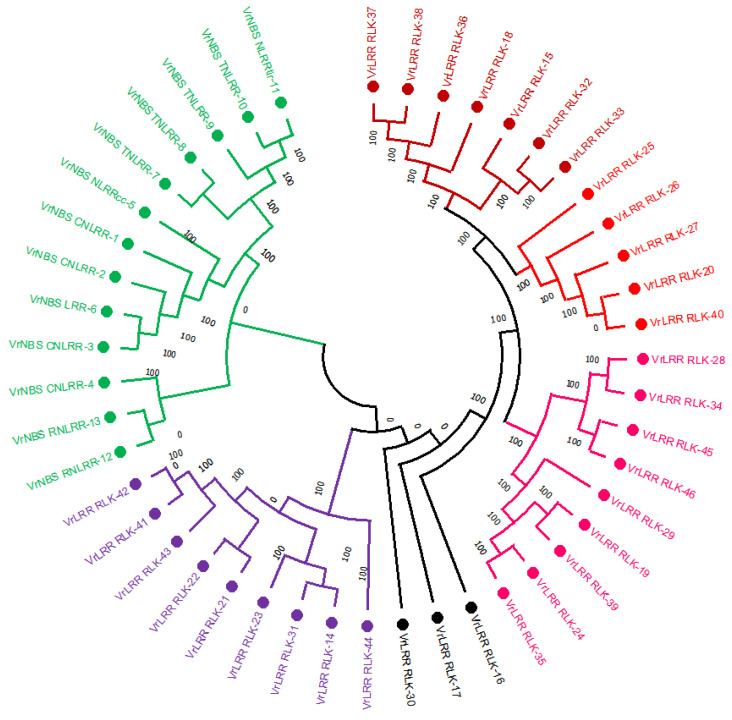
Phylogenetic tree of different LRR and LRR-RLK genes by adopting maximum livelihood model with bootstrap value *n* = 1000 using MEGA 11.0.

The phylogenetic tree of forty-six R-genes could be broadly divided into two clades (Figure 2), further subdivided into five sub-clades. Sub-clade-1 comprised 33 candidates, whereas sub-clade-2 comprised 13 R-candidates. Three genes, namely *VrLRR-RLK-16*, *VrLRR-RLK-17*, and *VrLRR-RLK-30*, were unique genes as they were distinctly grouped in separate sub-clades.

### 2.4. Gene Structure and Conserved Domain Prediction of Different R-Genes

**Figure 3:**
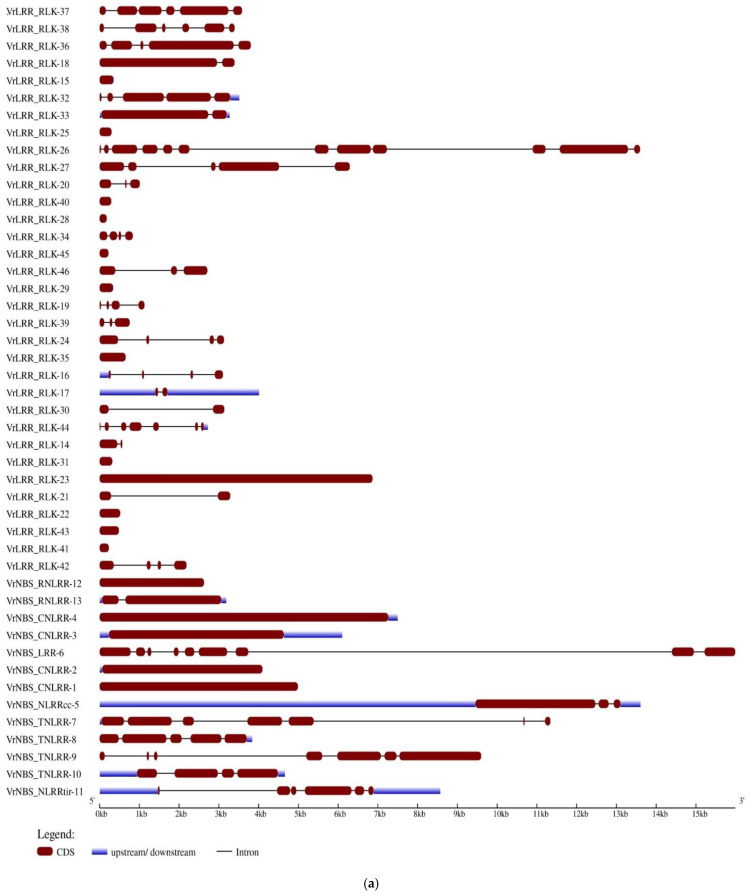

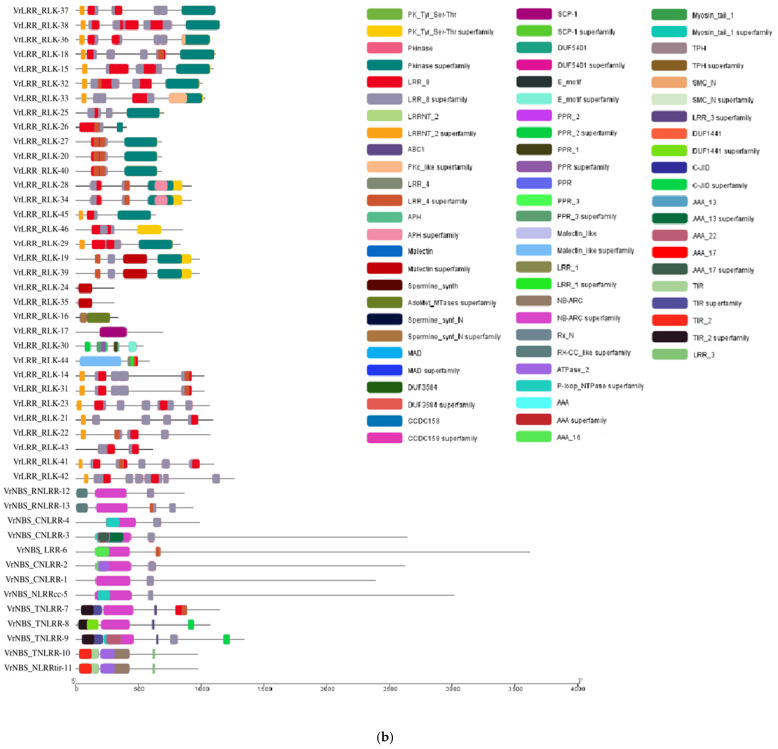
(**a**) Gene structure of different LRR and LRR-RLK genes. The blue line indicates the upstream/downstream regions; the red color indicates the CDS region; black lines denote introns. (**b**) Conserved domain architecture of different LRR and LRR-RLK genes.

Significant structural diversity was observed among the candidate R-genes retrieved in the present study. *VrNBS-LRR-6* was the longest gene at 18 kb, in which about 12 kb comprised the intronic region. This gene had the longest intronic region containing an untranslated region (UTR) of about 98% of its gene structure followed by *VrLRR_RLK-26*, *VrNBS_TNLRR-7*, and *VrNBS_TNLRR-9*. In the domain architecture, “PRR_3 superfamily” was identified in the majority of protein structures of different R-proteins (Figure 3). The “spermine synthase domain” was found only in the gene *VrLRR-RLK-44,* whereras the “LRR_8 domain” was found to be conserved in most of the R-proteins. Likewise, “Pkinase domain” was present in *VrLRR-RLK-37*, *VrLRR-RLK-38*, *VrLRR-RLK-36*, *VrLRR-RLK-46*, *VrLRR-RLK-28*, *VrLRR-RLK-34*, *VrLRR-RLK-19*, and *VrLRR-RLK-39*. Additionally, the “TPH superfamily” was present in two candidates, i.e., *VrNBS-TNLRR-10* and *VrNBS-NLRR-11*.

### 2.5. Motif Analysis of LRR and LRR-RLK Proteins

**Figure 4:**
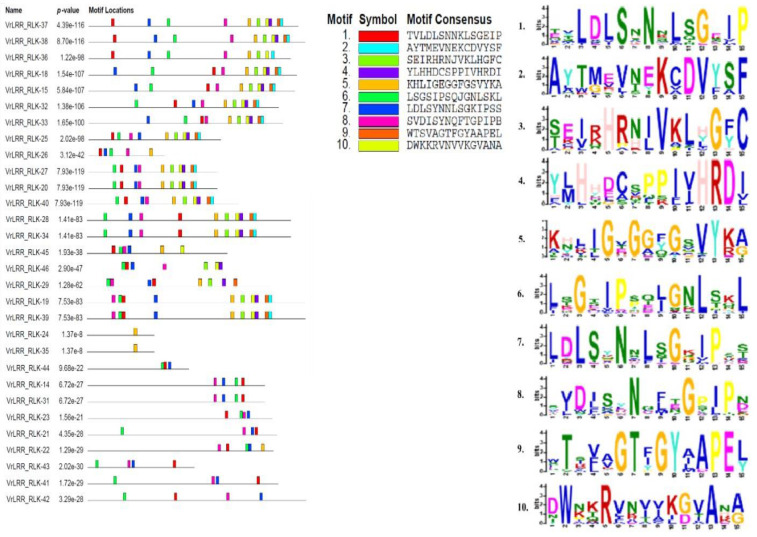

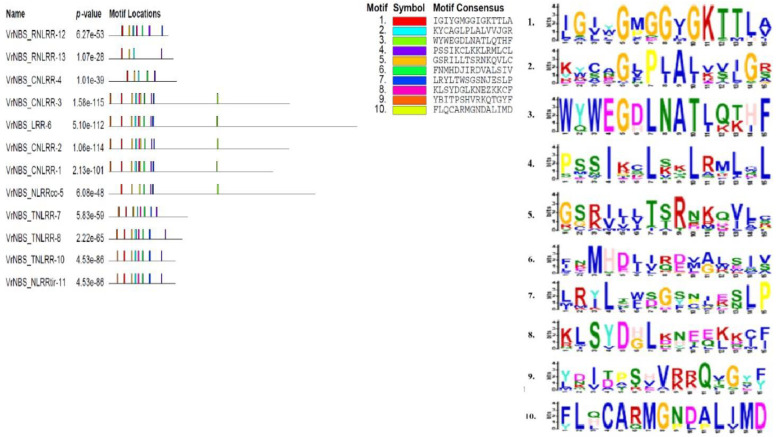
Motif analysis of *VrNBS-LRR* encoding genes.

The motifs for different LRR and LRR-RLK proteins were constructed in the MEME suite online software (https://meme-suite.org/meme/; accessed on 20 October 2024) with widths in the range of 5 to 50. The lengths of the motifs are indicated in Figure 4. The Pkinase domain contained conserved motifs (motifs 4, 5, 6, 8, 3, and 10). LRR_8 (Leucine-rich repeats 8), the LRR_8 superfamily (Leucine-rich repeats 8 superfamily), the LRRNT_2 (Leucine-rich repeats N-terminal) superfamily, the LRR_4 superfamily, and LRR_3 had M12, M1, M14, M9, M13, M2, M15, and M11 as conserved regions. All other domains, like spermine synthase and ATPase, did not occur frequently. Hence, motif conservation of these domains cannot be predicted accurately. The TIR_2 superfamily was found in the following R-proteins: VrNBS_RNLRR-12, VrNBS_RNLRR-13, VrNBS_CNLRR-4, VrNBS_LRR-6, VrNBS_CNLRR-2, VrNBS_CNLRR-1, VrNBS_NLRRcc-5, VrNBS_TNLRR-7, VrNBS_TNLRR-8, and VrNBS_TNLRR-9; M12, M1, M7, M12, M1, M7, and M10 were found, a diverse range. Thus, understanding the distribution of various domains in LRR proteins and subsequent analysis of motifs can help in understanding their specific function. In the motif sequence logo, some letters are stacked above each other, representing the sequence conservation at that position, which is measured in terms of bits. The height of the individual symbol within the stack represents the relative frequency of that amino acid at the respective position. A narrow letter signifies gaps in the alignment of the sequence.

### 2.6. In Silico Expression Analysis

**Figure 5:**
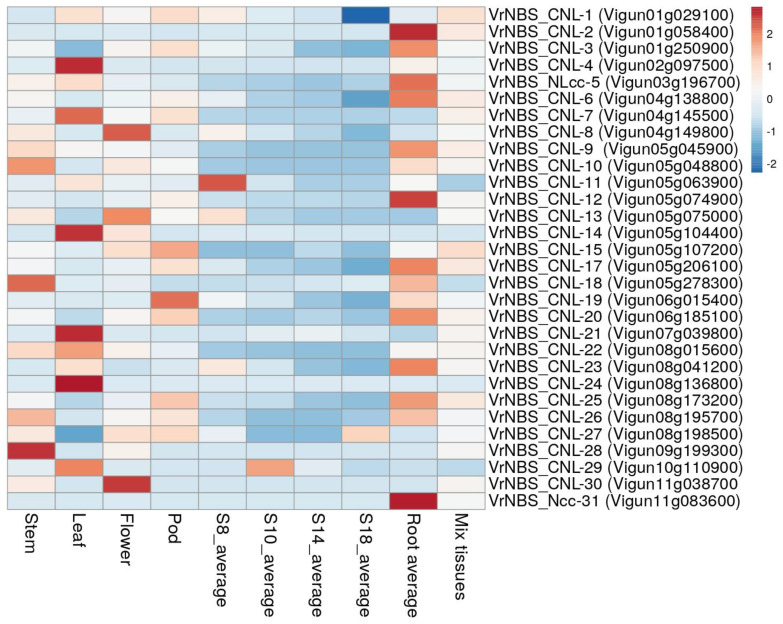
In silico gene expression profile of LRR and LRR-RLK candidates in different tissues of *Vigna unguiculata* using expression atlas. S8, S10, S14, and S18 are developing seeds.

All 42 candidate R-genes were subjected to BLASTn search against cowpea (*V. ungiculata*) as an expression atlas was present for this species in the LIS database (https://www.legumeinfo.org/; accessed on 20 October 2024). The sequences of *Vigna unguiculata* were retrieved and used for comparative expression analysis with *Vigna radiata* with the help of the CoNeKt tool in Legume Information Systems (https://conekt.legumeinfo.org/species/view/4; accessed on 20 October 2024), and a heatmap was created (https://conekt.legumeinfo.org/heatmap/; accessed on 20 October 2024) (Figure 5), illustrating the prediction of the expression of various characteristics in *Vigna radiata* in comparison to its orthologous genes in *Vigna unguiculata*.

The different rows show various R-genes, and columns represent expression in different organs of the plant. In the stem, the maximum expression of 34.63 was shown by the gene *VrNBS_CN-22,* followed by *VrNBS_CN-10* (16.95) and *VrNBS_CN-10* (16.49); the minimum expression of the respective genes is represented by green blocks. In the leaf, *VrNBS_CN-22* showed the maximum expression of 45.10, which is depicted by red color; the second largest expression was shown by *VrNBS_CNL-4,* showing expression of 40.45, and the minimum expressions were seen in *VrNBS_CN-30*, *VrNBS_Ncc-31*, *VrNBS_CN-28*, *VrNBS_CN-16*, and *VrNBS_CNL-2,* which had no expression. *VrNBS_CN-22* showed the maximum expression (24.31) in the flower. The range of gene expression in the pod was from 0 to 24.31 in *VrNBS_CNL-3* and *VrNBS_CNL-2*, respectively. In the case of roots, the maximum expression was seen in *VrNBS_CNL-3* (34.48). The genes *VrNBS_CN-24*, *VrNBS_CN-28*, *VrNBS_CN-29*, *VrNBS_CN-30*, *VrNBS_CN-3*, *VrNBS_CNL-2*, *VrNBS_CN-11*, *VrNBS_CN-12*, *VrNBS_CN-13*, *VrNBS_CN-14*, and *VrNBS_CN-16* showed minimum expression in all tissues, whereas *VrNBS_CN-3*, *VrNBS_CN-22*, and *VrNBS_CN-27* showed more than average expression.

### 2.7. Expression Profile of Identified Candidate R-Genes in Different Genotypes

**Figure 6:**
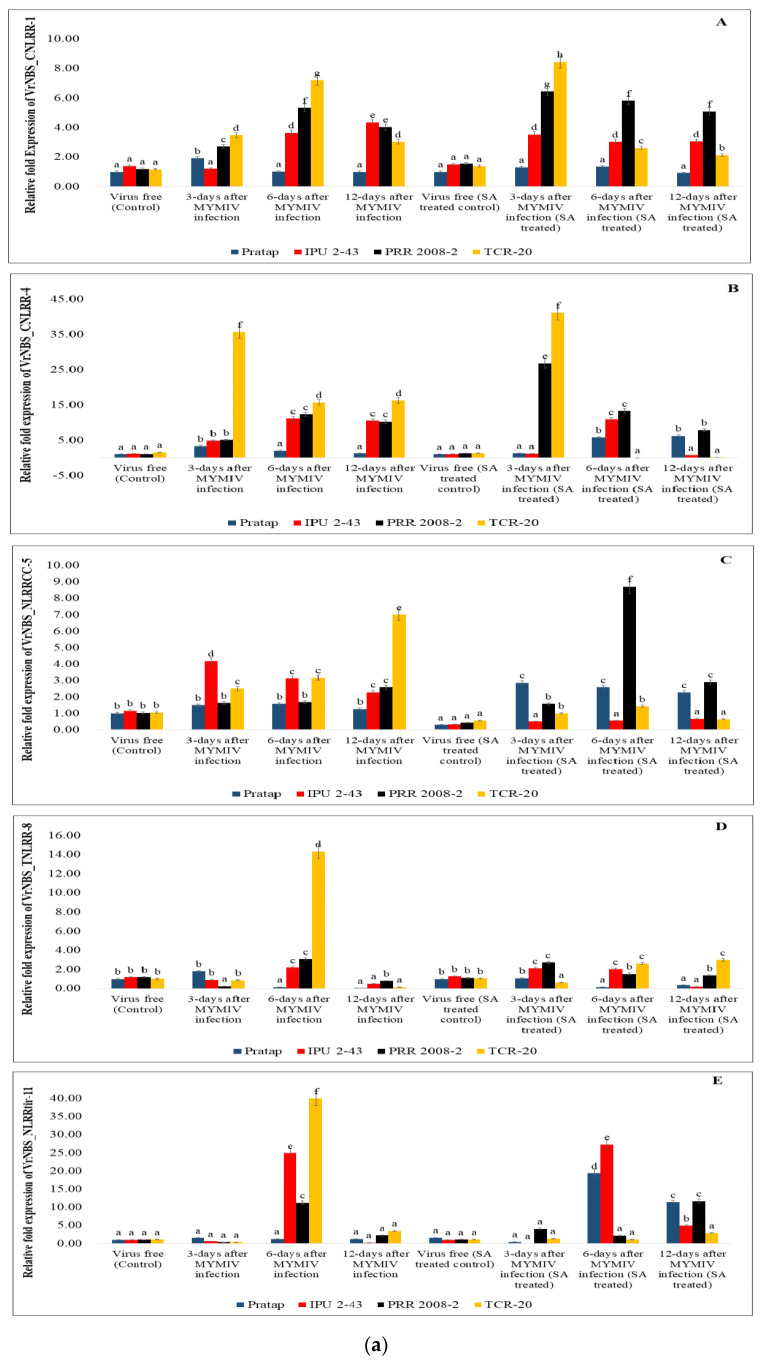

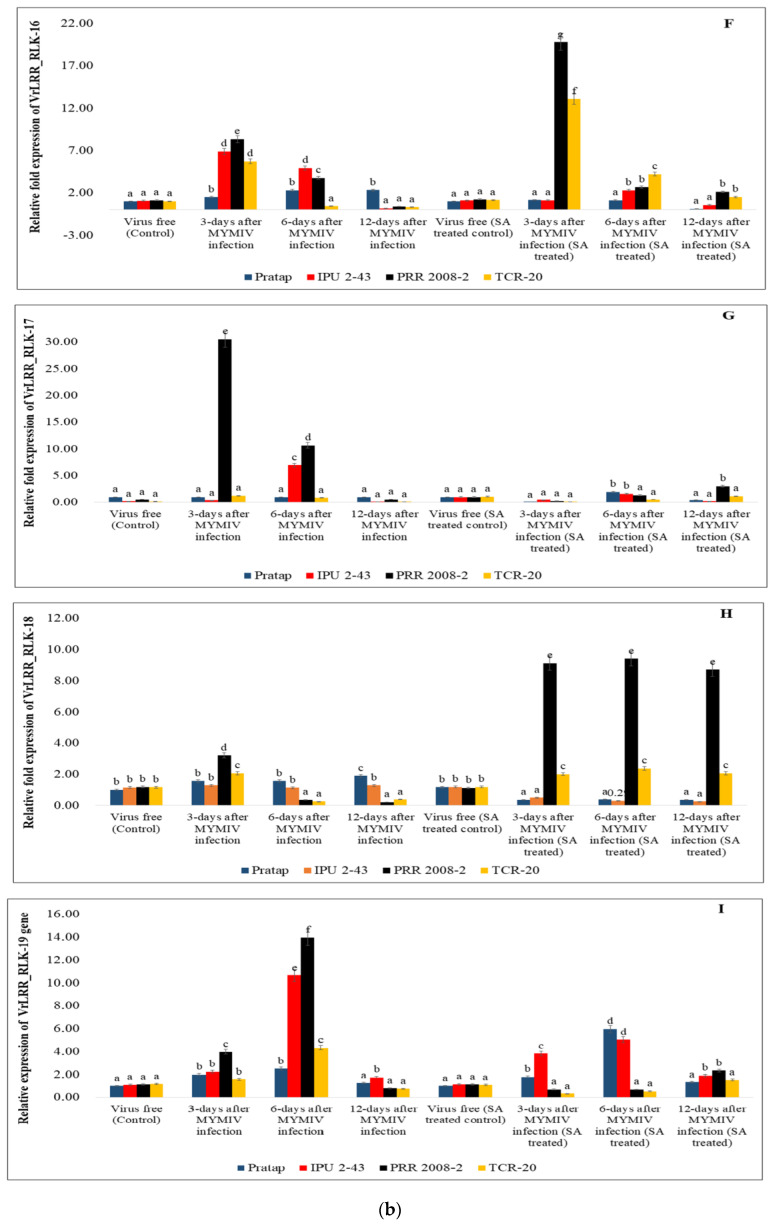

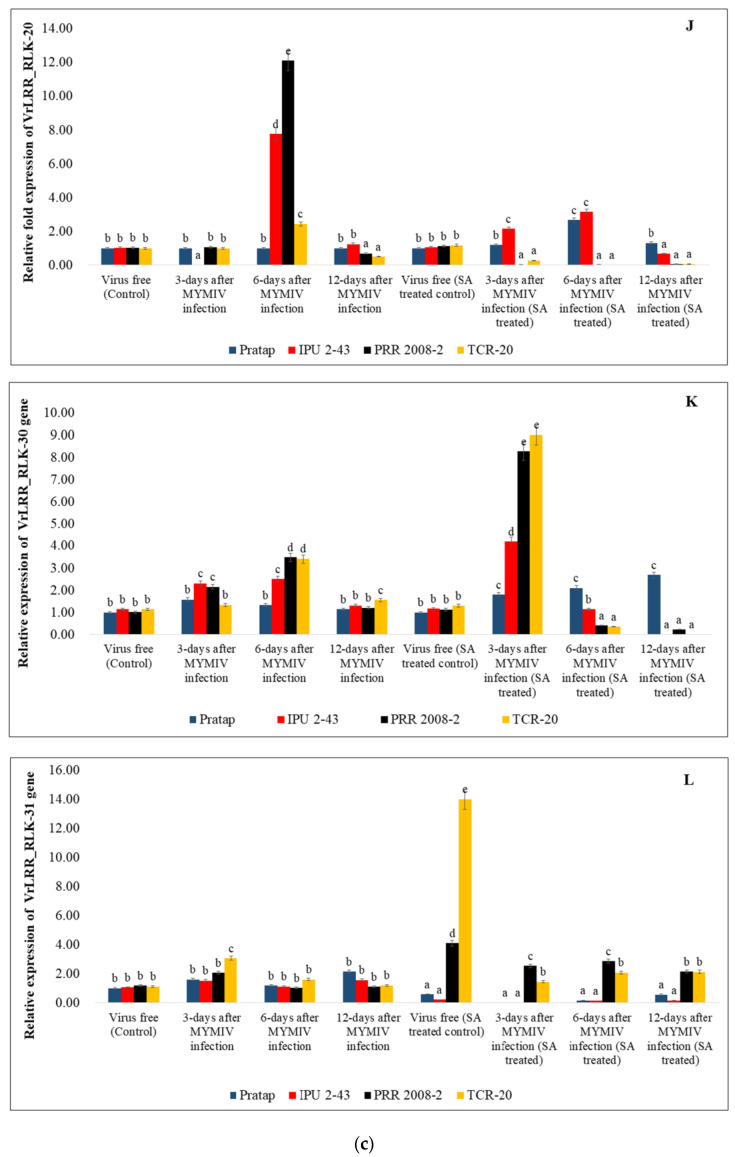
(**a**) qRT-PCR analysis of *LRR* candidate genes (**A**) VrNBS_CNLRR-1; (**B**) VrNBS_CNLRR-4; (**C**) VrNBS_NLRRcc-5; (**D**) VrNBS_TNLRR-8; (**E**) VrNBS_NLRRtir-11 under control and *MYMIV* infection in untreated and SA-treated genotypes. (**b**) qRT-PCR analysis of *LRR-RLK* candidate genes (**F**) VrLRR_RLK-16; (**G**) VrLRR_RLK-17; (**H**) VrLRR_RLK-18; (**I**) VrLRR_RLK-19 under control and *MYMIV* infection in untreated and SA-treated genotypes. (**c**) qRT-PCR analysis of *LRR-RLK* candidate genes (**J**) VrLRR_RLK-20; (**K**) VrLRR_RLK-21; (**L**) VrLRR_RLK-31 under control and *MYMIV* infection in untreated and SA-treated genotypes. The different letters in the graph showed significant differences among them.

A total of 12 representative candidate genes including LRRs and LRR-RLKs (Figure 6a–c) were subjected to gene expression profiling to understand the pattern of gene expression regulated by SA upon *MYMIV* infection. In *VrNBS_CNLRR-1*, all the genotypes showed variable expression in both control and SA treatment conditions. For *VrNBS_NLRRcc-5*, expression in all the genotypes was noticed in the control condition, but under the SA treatment conditions, the level of expression was increased 9-fold in PRR-2008-2 at 6-DAI, and at the rest of the time points, the expression was similar for all four genotypes. The expression of VrNBS_TNLRR-8 was also found to increase in SA treatment conditions in comparison to control conditions. In the case of VrNBS_NLRRtir-11, expression was seen at 6-DAI in IPU2-43, PRR-2008-2, and TCR-20 under control conditions, but after SA treatment, expression was seen in Pratap at 6-DAI (20-fold) and 12-DAI (15-fold). For VrLRR-RLK-16, expression reached the maximum at 3-DAI for all the genotypes but decreased at 6-DAI and 12-DAI under controlled conditions; however, for VrLRR-RLK-16 (SA-treated), expression was decreased. For VrLRR_RLK-30 (H_2_O), all the genotypes showed expression at all time points, but under salicylic acid treatment conditions, it decreased after 3-DAI in all four genotypes in VrLRR_RLK-30 (SA). For the VrLRR_RLK-18 gene, Pratap, IPU2-43, PRR-2008-2, and TCR-20 all showed expression under H_2_O spraying conditions, whereas a similar relative expression (10-fold and 5-fold) was seen in PRR-2008-2 and TCR-20, respectively.VrLRR_RLK-31’s gene expression was observed in different genotypes at all time points, but no appreciable level of expression was seen in treatment conditions.

### 2.8. Predicted Structure Analysis of LRR Receptors

**Figure 7:**
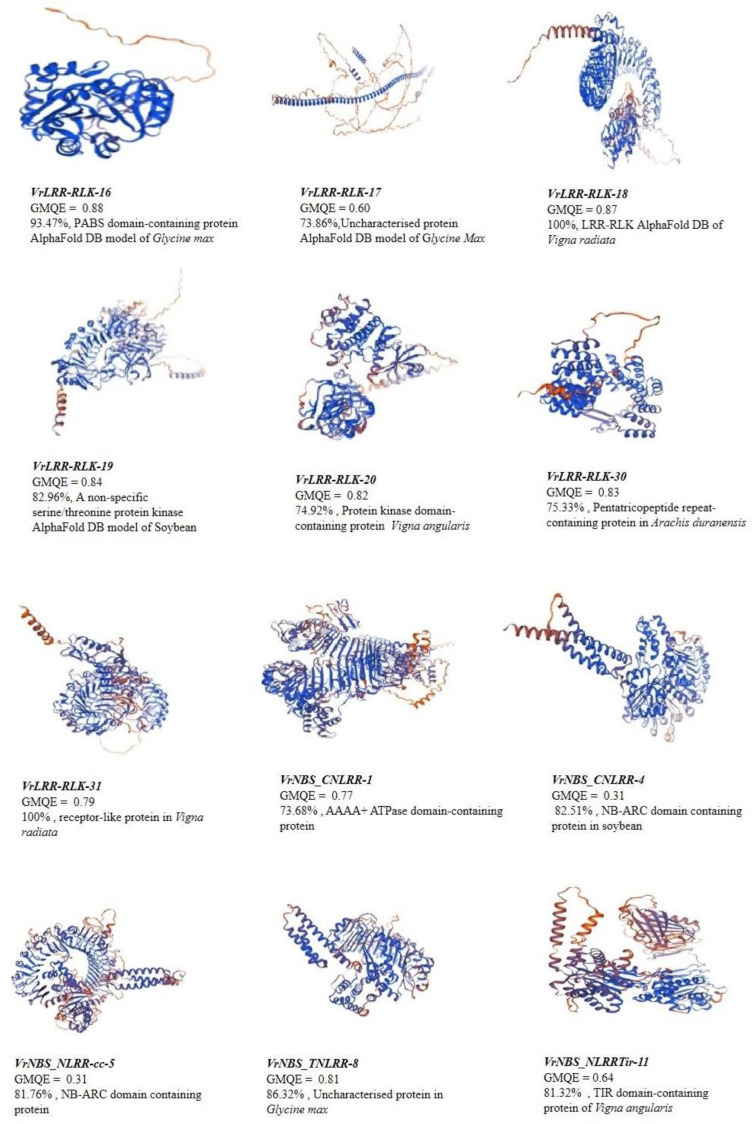
Protein structure prediction of selected LRR and LRR-RLK candidate genes.

Gene sequences encoding PABS domain-containing proteins were analyzed using Alpha Fold DB to model their structures, providing insights into sequence identity, organism origin, oligomeric state, and GMQE (Global Model Quality Estimation) scores (Figure 7, Table S1). *VrLRR_RLK-16* showed a 93.18% sequence identity with *Glycine max* (soybean), with a monomeric form and a high GMQE score of 0.88. A second model for this gene, Thermospermine Synthase (*MtTSPS*), exhibited 91.9% identity with *Medicago truncatula*, with a GMQE of 0.81. VrLRR_RLK-17 matched an uncharacterized protein from *Glycine max* at 72.62% identity and a GMQE of 0.60. *VrLRR_RLK-18* demonstrated 100% identity with a probable LRR receptor-like protein kinase from *Vigna radiata var. radiata* and had a GMQE of 0.87. *VrLRR_RLK-19*, another LRR-RLK, showed 81.96% identity to a non-specific serine/threonine protein kinase from *Glycine max*, with a GMQE of 0.84. *VrLRR_RLK-30* was aligned with a pentatricopeptide repeat-containing protein from *Arachis duranensis* at 75% identity and a GMQE of 0.83. *VrLRR_RLK-31* showed 100% identity to a receptor-like protein isoform X1 from *Vigna radiata var. radiata*, with a GMQE of 0.79. For NBS-LRR proteins, *VrNBS_CNLRR-1* exhibited 72.3% identity with an AAA+ ATPase domain-containing protein from *Glycine max* and a GMQE of 0.69. *VrNBS_CNLRR-4* matched a TIR domain-containing protein from *Glycine max* at 79.02% identity and a GMQE of 79.05%. Finally, VrNBS_NLRRtir-11 had 82.51% identity with an NB-ARC domain-containing protein from *Glycine max* and a GMQE of 0.76.

### 2.9. Protein–Protein Interaction

**Figure 8:**
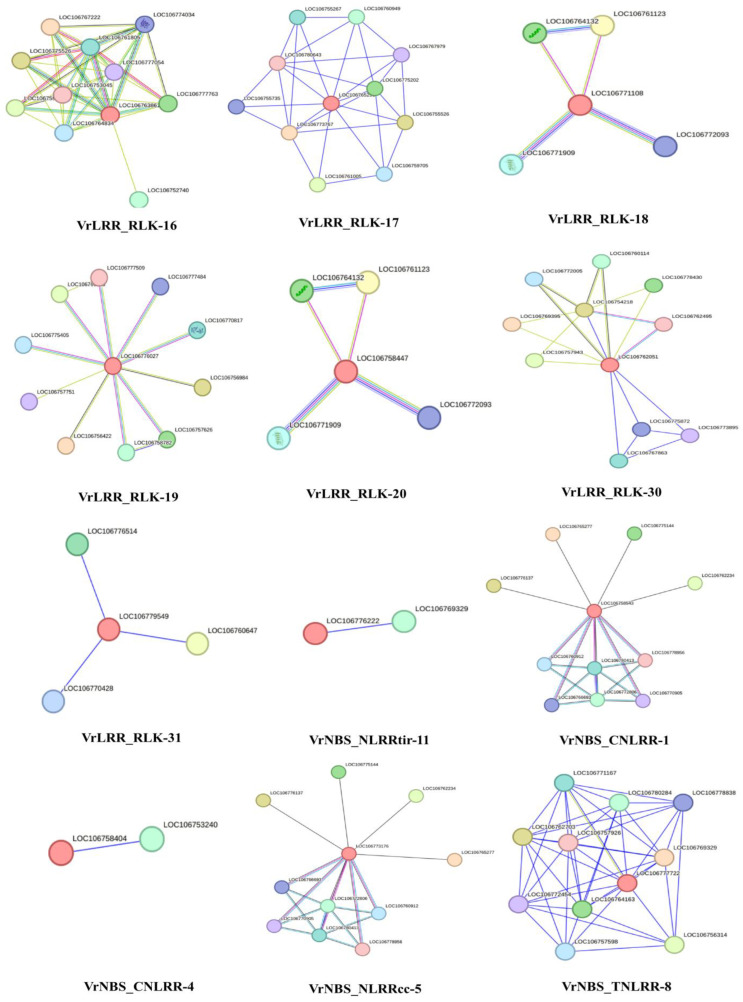
Protein–protein interaction network based on STRING database of the selected genes.

Protein–protein interactions for all the selected genes were predicted using the string database (Figure 8, Table S2). The protein–protein interaction analysis of *Vigna radiata* LRR and NLR gene families revealed various key interacting partners. The protein VrLRR_RLK-16 showed interactions with multiple S-adenosylmethionine decarboxylase proenzymes, such as LOC106767222, LOC106775526, LOC106759397, and LOC106777763, each with a high interaction score of 0.837 or 0.831. Additionally, interactions were observed with proteins involved in root development (LOC106752740) and various decarboxylases, including ornithine (LOC106761805) and glutamate (LOC106764834) decarboxylases, with scores above 0.780. VrLRR_RLK-17 interacted with several uncharacterized proteins (e.g., LOC106773767, LOC106775202) and a pectinesterase inhibitor, albeit with lower interaction scores ranging between 0.502 and 0.468. Similarly, VrLRR_RLK-18 interacted with proteins like BRI1 kinase inhibitors (LOC106761123, LOC106764132) and somatic embryogenesis receptor kinases, although the scores for these interactions were modest, around 0.446. VrLRR_RLK-19 showed interactions with transcription factors, such as Myb-related protein 305-like (LOC106756422), transcription factor JAMYB-like (LOC106756984), and calcium-binding proteins, including calmodulin-3 (LOC106757626) and calmodulin-like protein 11 (LOC106770817), with interaction scores between 0.579 and 0.567. Furthermore, VrLRR_RLK-30 and VrLRR_RLK-31 interacted with proteins involved in biosynthesis and stress response, such as phosphatidylinositol glycan anchor biosynthesis protein (LOC106769395), kinesin-like proteins, and TMV resistance proteins, with scores ranging from 0.325 to 0.479. In the NBS family, VrNBS_CNLRR-1 and VrNBS_NLRRcc-5 exhibited interactions with sulfate transporters (LOC106765277, LOC106776137) and serine/threonine protein kinases (LOC106760912, LOC106766693) with interaction scores around 0.237 and 0.200. Lastly, VrNBS_TNLRR-8 and VrNBS_NLRRtir-11 displayed interactions with proteins like WVD2-like 4 isoform X1 and F-box/LRR-repeat proteins, with scores between 0.330 and 0.412. These interactions provide valuable insights into the functional roles of these proteins in stress response, development, and metabolic regulation in *Vigna radiata*. Thus, the protein–protein interaction analysis of *Vigna radiata* LRR and NLR gene families highlights significant interactions with proteins involved in various biological processes, such as decarboxylation, kinase activity, calcium binding, and sulfate transport. VrLRR_RLK proteins exhibited strong interactions with S-adenosylmethionine decarboxylase proenzymes, root meristem growth factors, and BRI1 kinase inhibitors, indicating their role in developmental and stress response pathways. Similarly, VrNBS proteins interacted with key components like sulfate transporters and serine/threonine kinases, suggesting their involvement in regulatory and stress mechanisms. The diverse range of interactions underscores the functional complexity of LRR and NLR proteins in *Vigna radiata*, with implications for understanding stress tolerance, growth, and metabolic regulation.

## 3. Discussion

YMD is the most devastating disease of *Vigna* species [24] as it is caused by three different viruses, *MYMV*, *MYMIV*, and *HgYMV*, whose genome sequences are very close to each other [24]. Hence, proper diagnosis of the pathogen causing YMD is essential before planning any mitigation strategies, including resistance breeding. We already identified the YMD-causing virus as *MYMIV* in the central zone of India, where our experimental plot is situated [4]. We also performed comprehensive screening of mungbean (*Vigna* radiate), urdbean (*Vigna* mungo), and its wild relatives, including progenitors and non-progenitors, and identified potential donors for *MYMIV* resistance [4,25]. Crop wild relatives (CWRs) are known to be reservoirs of several important genes, especially for stress tolerance. The plant materials selected from these previous studies were IPU 02-43 (*MYMIV* resistant cultivar), PRR 2008-2 (*MYMIV* resistant wild non-progenitor), TCR-20 (*MYMIV* resistant wild non-progenitor) and Pratap (*MYMIV* susceptible cultivar). SA (100 µMol) was used for foliar priming for the induction of resistance in HS and HR genotypes to understand the molecular responses of LLR and LRR-RLK genes in unprimed and primed plant species. Several workers reported SA as a signaling molecule in the induction of resistance [26,27,28]. Iqbal et al. [29] reported that SA activates various antioxidants, elicitors, pathogenesis-related (PR) genes, and systemic acquired resistance (SAR) pathways and triggers programmed cell death to improve resistance. Wang et al. [30] reported that SA significantly enhances the level of endogenous SA and finally improves the level of resistance in plants.

LRR and LRR-RLK genes play a significant role in signal transduction upon interacting with pathogen effectors, which triggers effector-mediated immunity, as reported by Lolle et al. [31]. It also acts as a signaling receptor [32]. Due to the unavailability of a chromosome-level assembly of blackgram, we retrieved 46 LRR and LRR-RLK genes in *Vigna radiata*, a close relative of *Vigna mungo*. Upon analyzing the gene sequences, it was observed that 13 of these sequences contained an NBS-LRR domain, while the remaining 33 contained an LRR-RLK domain. The 13 NBS-LRR genes could be classified into four categories as 4 CNLs, TNL, 2 RNLs, and 3 NLRs. These genes exhibited a central NBS domain, with the C-terminal region containing the LRR domain, and N-terminal variations as reported by Meyers et al. [33]. Furthermore, a coiled-coil structure was identified at the N-terminus in some of the genes [34]. Yuan et al. [35] reported 234 LRR-RLK candidates in *Arabidposis thaliana*, and Cheng et al. [36] reported 437 LRR-RLK candidates in *Saccharum* spp., which were relatively higher than those in *Vigna radiata.* The RLK family was characterized based on amino acid sequences located in the extracellular domain (ECD) of the N-terminal region, as described by Shiu and Bleecker [37]. Phylogenetic analysis revealed that all forty-six LRR genes could be grouped into eight distinct sub-clades. The divergence among these genes was thought to be due to their roles in response to various biotic and abiotic stresses. Mapping the physical locations of these genes on the *Vigna radiata* genome showed that they were randomly distributed across all 11 chromosomes, indicating a lack of collinearity between the identified genes and those previously known on the chromosomes.

Gene structure analysis revealed a significant variation in intron and exon numbers, which was attributed to evolutionary divergence. Protein domain structures, derived from the conserved domain database (CDD), showed the presence of various LRR subfamilies within the identified genes. The LRR_8 superfamily was found to be the most conserved, present in nearly all genes. In 16 of the LRR genes, conserved domains such as Pkinase, Pkinase_Tyr, and the Pkc_like superfamily were identified, which are associated with leucine-rich repeat receptor kinases (LRR-RKs). LRR-RKs constitute a large family of plant receptor kinases that play crucial roles in plant development and immunity. The LRR domain is known to serve multiple functions, including effector recognition, acting as an auto-inhibitory domain, and preventing the auto-activation of downstream signaling pathways. Furthermore, the TIR_2 superfamily was conserved in 10 of the R-genes, while other genes exhibited various domain types, reflecting the diversity among the identified proteins. The TIR domain, which is part of the TIR-NB-LRR resistance protein family, functions as a signaling domain involved in triggering cell death. This domain is located within conserved regions, known as TIR2 and TIR3, and is also found in loosely conserved sequences between these regions [38].

Motif prediction indicated a high level of conservation in leucine-rich repeats (LRRs). As shown in Figure 4, from the MEME analysis, motifs 1, 2, 5, 6, 7, 9, and 11 were identified as leucine-rich compared to the others. According to Ng and Xavier [39], most of the LRR domains consisted of 2 to 45 leucine-rich repeats, each repeat comprising about 20 to 30 residues. Structurally, LRR domains form an arc or horseshoe shape, with parallel β-strands on the concave face and more variable secondary structures, such as helices, on the convex face. The presence of common motifs across different proteins suggests a degree of functional redundancy, as also noted by Liu et al. [40]. Leucine-rich sequence logos were proposed as an effective way to visualize consensus sequences and diversity in aligned sequences. These logos help in predicting functional protein units. In the sequence logos, vertically stacked letters represent sequence conservation, measured in bits, with the height of each letter reflecting the relative frequency of that amino acid at a specific position. Narrower letters indicate gaps in the sequence alignment. The LRR is characterized by a conserved pattern of hydrophobic leucine residues. These LRR domains exhibit broad interaction surfaces, capable of accommodating high variability, providing a versatile platform for diverse immune receptors. As described by Padmanabhan et al. [41], LRRs enable protein–protein interactions that are essential for their dual function as sentries and activators of defense responses. Comparative gene expression analysis is a method that helps in learning about the presence/absence of functionally related genes across the species [42]. This was achieved by identifying the orthologous gene of *Vigna radiata* in *Vigna unguiculata* through NCBI BLAST and the subsequent creation of a heatmap. Through the heatmap, we estimated the expression level of a particular *Vigna radiata* gene in different tissues of the plant and through comparison with *Vigna unguiculata*. The *VrNBS_CNL-3* gene had the maximum level of expression of all the genes. *VrNBS_CN-22* showed expression in leaf, stem, flower, pod, root, and mixed tissues. However, based on in silico analysis, *VrNBS_CN-16*, *VrNBS_CN-24*, and *Vr_NBS_Ncc-31* showed a minimal level of expression in all the tissues studied. This is just a prediction based on bioinformatics stools, but it may help during functional validation of the expression of different genes in different tissues. Only 31 genes of *Vigna unguiculata* were found to be orthologous to the *Vigna radiata* genome. This may be due to the duplication of genes in *Vigna unguiculata.*

The expression of representative R-gene candidates from different clades indicated that there were significant variations in the expression pattern in both SA-primed and unprimed plants. *VrNBS_CNLRR-1* is significantly expressed in both SA-treated and untreated plants. *VrNBS_TNLRR-8*, *VrLRR_RLK-20*, *VrLRR_RLK-17*, and *VrLRR_RLK-19* showed appreciable expression upon SA treatment. Kundu et al. [43] performed transcriptome analysis in blackgram and identified the NB-LLR candidates modulating YMD resistance. Maiti et al. [44] reported two “R-genes”, YR-4 and CYR-1, as being linked with YMD resistance. Purwar et al. [38] also characterized the NBS-LRR gene family in mungbean and suggested a significant up-regulation of NBS-LRR candidates in resistant genotypes upon *MYMIV* infection. Khoshru et al. [45] reported that SA acts as a signaling molecule for SAR, contributing to the activation of the defense mechanism by activating the antioxidant-related genes against pathogen attack. It also stimulates SOD, GPX, and phenolics and prevents ROS-mediated cell death [6]. Kumar et al. [46] reported the higher expression of SOD and WRKY genes in YMD-resistant genotypes compared to susceptible ones. Singh et al. [25] reported the increased response of superoxide anion scavenging activity (SASA), ferric reducing antioxidant power (FRAP), and 2,2-diphenyl-1-picryl hydrazyl (DPPH) free radical scavenging activity in resistant genotypes of blackgram upon YMD infection, whereas reduced expression of CAT and higher activity of NADPH oxidase promoted ROS production in a susceptible blackgram cultivar. This indicates that SA successfully modulates ROS as a resistance response by activating SA-related genes, regulating antioxidant genes and ROS homeostasis. Further, the mystery behind these differential expressions of candidate genes can be made more clear after functional validation of these genes. Functional validation will help us to correlate the factors that lead to differential expression at different time points and under different conditions. Through the SWISS-MODEL, we may conclude that their structural integrity was maintained during evolution, which is essential for understanding their function. The closer the GMQE value is to 1, the better the quality of the structure that is predicted [47]. Through our analysis, we can conclude that most of the predicted spatial structure showed similarity with template proteins of soybean, wild peanut, and *Vigna* spp. This dataset includes receptor-like kinases (LRR-RLKs) and resistance proteins (NBS-LRRs) from *Vigna radiata* with varying degrees of sequence identity to homologs in *Glycine max*, *Medicago truncatula*, and *Arachis duranensis*. The proteins are involved in critical plant functions such as pathogen recognition, stress responses, and growth regulation, highlighting their importance in plant immunity and signaling pathways. The GMQE scores provide insights into the structural confidence of these models, with higher scores indicating greater model reliability [48,49]. The comprehensive analysis of the LRR and LRR-RLK gene family revealed that significant structural diversity was observed within the family. Candidates such as *VrNBS_TNLRR-8*, *VrLRR_RLK-20*, *VrLRR_RLK-17*, and *VrLRR_RLK-19* were significantly regulated during the *MYMIV* infection in both SA-primed and unprimed *Vigna* species. These genes were related to the translation of R-proteins, serine/threonine protein kinases, TMV resistance, and F-box/LRR proteins, indicating their significant involvement in modulating *MYMIV* resistance.

## 4. Materials and Methods

### 4.1. Plant Materials and Stress Treatment

The plant material comprised four genotypes, including two cultivated and two wild species. Pratap Urd-1 was characterized as highly susceptible to YMD, whereas IPU 02-43 was characterized as highly resistant, and both belong to *Vigna mungo*. In addition, two wild non-progenitors, i.e., TCR-20 (HR) and PRR 2008-2 (HR), belonging to *Vigna glabrescens* and *Vigna umbellata,* respectively, were used in the present study. These genotypes were grown in two different sets. The first set was kept in an insect-proof net house to avoid exposure to white fly (*Bemisia tabaci*), whereas the second set was kept in a shaded and netted greenhouse at Banda University of Agriculture and Technology, Banda (24°53′ latitude and 25°55′ longitude), during the rainy season of 2023. Further, to ensure a sufficient amount of *MYMIV* inoculums on the plants, the plants at the seedling stage (15 days after germination) were subjected to force-feeding by whitefly being reared on susceptible plants in the wild garden. A total of 20 whiteflies per plant were introduced into the cage and were allowed to feed for 48 h. Both sets (control and YMD) were subjected to a foliar spray of water or SA, 100 uMol, 48 h prior to YMD infection. At 0 (as control), 3, 6, and 12-DAI, the leaf samples were plucked and immediately frozen in liquid nitrogen for further analysis.

### 4.2. RNA Extraction and cDNA Synthesis

In total, 100 mg of frozen leaves of each genotype at selected time points was homogenized using tissuelyser-II (Qiagen, Hilden, Germany) at a frequency of 30/s for 2 min. RNA was extracted using a RNeasy Mini Kit (Qiagen) following the manufacturer’s instructions, and the extracted RNA was quantified using a micro-volume spectrophotometer (QIAExpert System, Qiagen). The DNase treatment was performed to remove the DNA contaminants from the extracted RNA. Subsequently, 1 µg of RNA was reverse-transcribed by using a RevertAid First Strand cDNA Synthesis Kit (Thermo Fisher Scientific, Bangalore, India). The synthesized cDNA was then normalized at 50 ng/µL and utilized for qRT-PCR analysis.

### 4.3. Genome-Wide Identification, Characterization, and Distribution of LRR and LRR-RLK Candidates

The publicly available genome assembly of mungbean [50] was used to retrieve the R-proteins using the Pfam id (PF00931). The data were retrieved from the Legume Information System (https://legumeinfo.org/; accessed on 15 April 2024) with HMMER V.3 [51]. The results were, in turn, confirmed using the National Center for Biotechnology Information (NCBI) conserved domain database (http://www.ncbi.nlm.nih.gov/Structure/cdd/wrpsb.cgi; accessed on 15 April 2024) using an *e*-value of 0.01 as well as the Pfam database (http://pfam.sanger.ac.uk/; accessed on 15 April 2024). The CC motifs were predicted through the COILS program (https://toolkit.tuebingen.mpg.de/tools/hhblits; accessed on 15 April 2024). The VrNBS-LRR and VrLRR-RLK encoding genes were categorized based on the arrangement of the protein domains. Further, the physical positions (bps) of the VrNBS-LRR and VrLRR-RLK encoding genes were acquired from the LIS database and mapped on to their corresponding chromosomes using MapChart version 2.3 [52]. The genes present in the scaffold could not be assigned to the chromosome due to the non-availability of their correct positions.

### 4.4. Evolutionary Tree Analysis

Multiple alignments of the deduced amino acid sequences were performed using ClustalW [53], and the phylogenetic tree was constructed using the maximum likelihood (ML) method [54] based on the Jones–Taylor–Thornton (JTT) matrix-based model with 1000 bootstrap replications using MEGA 11.0 [55].

### 4.5. Gene Structure, Domain, and Conserved Motif Prediction

*LRR* and *LRR- RLK* candidate genes were checked for intron and exon structure using GSDS software V.2.0 based on comparisons among the full-length genome sequences and protein-coding sequences [56]. LRR and LRR–RLK protein domains and active motif function were analyzed in the Pfam database (http://pfam.xfam.org; accessed on 15 April 2024) using NCBI-CDD [57] and constructed via TB tools-II software (v2.142) (https://github.com/CJ-Chen/TBtools-II/; accessed on 15 April 2024). The web-based motif identification server MEME-Suite (Multiple Em for Motif Elicitation, http://meme-suite.org/meme/; accessed on 15 April 2024) was used to detect potential motifs with a motif width between 6–15.

### 4.6. In Silico Expression

For in silico comparative expression analysis, BLAST was performed on the gene sequences against *Vigna unguiculata* and they were subsequently downloaded. Through Conekt Comparative Expression (https://conekt.legumeinfo.org/species/; accessed on 15 April 2024), a heat map was generated (https://conekt.legumeinfo.org/heatmap/, accessed on 15 April 2024). The generated heat map was used to analyze the expression of LRR and LRR-RLK candidates in different tissues.

### 4.7. Selection of Candidate Genes, Primer Design, and qRT-PCR Analysis

The primers were designed based on VrNBS-LRR and LRR-RLK sequences using the primer-blast tool (https://www.ncbi.nlm.nih.gov/tools/primer-blast/; accessed on 20 April 2024) and were subsequently employed for expression analysis (Table 2). The criteria for primer designing were a GC content >60%, average annealing temperature of 60 °C, and expected amplicon size of about 150–250 bp.

In total, 10 µL of 2X Dream Taq green q-PCR master mix (Thermofisher Scientific, Waltham, MA, USA), 1 µL of 10 pmol each of the forward and reverse primers (Europhins India Pvt. Ltd., Bangalore, India), 6 µL of nuclease-free water, and 2 µL of cDNA were used to prepare the reactions. The fast-cycling two-step program was run with 2 min of initial denaturation at 96 °C; 40 cycles of 15 s denaturation at 96 °C; and 60 s annealing and extension at 60 °C. *Actin* was used as an internal control. The reactions were carried out in a QuantStudio 5 Real-Time PCR machine (Thermofisher Scientific, Waltham, MA, USA). Three biological replicates were taken, and from them, two technical replicates were used for analysis. Thereafter, the relative expression of the genes were analyzed by the delta–delta CT method [58]. Duncan’s Multiple Range Test (DMRT) was performed to test for significance.

### 4.8. Predicted Protein Structure, Interaction Network, and Co-Expression Network Construction

The selected genes were analyzed to predict the spatial structure of LRR or LRR-RLK proteins with the help of the online software SWISS-MODEL (https://swissmodel.expasy.org/; accessed on 20 April 2024). The interacting networks of LRR and LRR-RLK proteins were integrated into the STRING [59] (https://www.string-db.org/; accessed on 20 April 2024) software, followed by an export of the co-expression network data from STRING, which were further calculated using Microsoft Excel 2019.

## Figures and Tables

**Table 1:** Characterization of LRR and LRR-RLK encoding genes.

Alias Name	Gene ID	Start	End	Genomic Length (bp)	CDS Length (bp)	LG	Protein Length (AA)	Molecular Weight (KD)	pI	Exons
VrNBS_CNLRR-1	Vradi04g06660	14210891	14220840	9949	4989	4	1662	191.81	5.83	5
VrNBS_CNLRR-2	Vradi04g06670	14251005	14255633	4628	4097	4	1342	155.21	5.76	4
VrNBS_CNLRR-3	Vradi04g06680	14272269	14285978	13709	6105	4	1464	169.18	5.66	8
VrNBS_CNLRR-4	Vradi04g06840	14628767	14647415	18648	7498	4	2420	272.73	6.5	23
VrNBS_NLRRcc-5	Vradi09g04000	5436962	5450570	13608	3772	9	1093	130.68	6.09	5
VrNBS_LRR-6	Vradi09g07330	12141674	12157663	15989	3795	9	1264	145.8	5.57	9
VrNBS_TNLRR-7	Vradi0023s00600	1453281	1464622	11341	3643	Scf	1199	137.21	7.01	7
VrNBS_TNLRR-8	Vradi02g09230	14138816	14142451	3635	3222	2	1057	121.51	6.65	5
VrNBS_TNLRR-9	Vradi07g04750	8980383	8989982	9599	4110	7	1369	155.74	8.38	7
VrNBS_TNLRR-10	Vradi10g01550	4532573	4537238	4665	3465	10	970	111.08	7.17	5
VrNBS_NLRRtir-11	Vradi06g03670	4063230	4071861	8631	5064	6	1687	191.8	5.36	10
VrNBS_RNLRR-12	Vradi05g17300	26439031	26441659	2628	2628	5	875	100.33	8.5	1
VrNBS_RNLRR-13	Vradi11g08410	9037552	9040739	3187	3012	11	940	107.32	9.09	2
VrLRR_RLK-14	Vradi0364s00070	221815	222387	573	489	scf	1024	115.02	5.46	1
VrLRR_RLK-15	Vradi04g08160	16479429	16479782	354	354	4	1097	120.46	7.02	1
VrLRR_RLK-16	Vradi05g23590	37053695	37056798	3104	369	5	337	38.66	5.72	1
VrLRR_RLK-17	Vradi06g16080	36338628	36342637	4010	192	6	691	78.29	8.44	1
VrLRR_RLK-18	Vradi08g04290	7932027	7935482	3459	3324	8	1107	122.49	6.06	1
VrLRR_RLK-19	Vradi10g09720	17290628	17291818	1191	507	10	988	109.79	5.79	1
VrLRR_RLK-20	Vradi11g09460	11258567	11259580	1014	564	11	680	76.27	6.54	1
VrLRR_RLK-21	Vradi01g00000986	14577697	14580983	3287	600	1	1092	123.68	5.82	1
VrLRR_RLK-22	Vradi01g00001365	23290961	23291479	519	519	1	1073	122.25	7.55	1
VrLRR_RLK-23	Vradi02g00003902	66843924	66850789	6866	6866	2	1066	120.69	5.04	1
VrLRR_RLK-24	Vradi03g00001007	15971710	15974841	3132	825	3	304	33.57	9.97	1
VrLRR_RLK-25	Vradi03g00001199	23258997	23259299	303	303	3	703	79.67	6.96	1
VrLRR_RLK-26	Vradi04g00000481	4153585	4167179	13595	5370	4	402	44.7	5.57	1
VrLRR_RLK-27	Vradi04g00000484	4174685	4180979	6295	2826	4	680	76.27	6.54	1
VrLRR_RLK-28	Vradi04g00003234	33378300	33378479	180	180	4	922	99.6	6.13	1
VrLRR_RLK-29	Vradi05g00000393	4807277	4807618	342	342	5	832	92.09	5.54	1
VrLRR_RLK-30	Vradi05g00000473	6026299	6029438	3140	513	5	532	59.48	6.74	1
VrLRR_RLK-31	Vradi05g00000709	12794518	12794838	321	321	5	1024	115.02	5.46	1
VrLRR_RLK-32	Vradi06g00001292	9691186	9694702	3517	2721	6	1010	111.86	9.08	1
VrLRR_RLK-33	Vradi06g00001294	9706565	9709835	3271	3048	6	1033	112.78	5.93	1
VrLRR_RLK-34	Vradi07g00000022	710126	710960	835	633	7	922	99.6	6.13	1
VrLRR_RLK-35	Vradi07g00001333	15944451	15945107	657	657	7	304	33.57	9.97	1
VrLRR_RLK-36	Vradi07g00001343	16073870	16077673	3804	3210	7	1073	118.56	6.72	1
VrLRR_RLK-37	Vradi07g00001347	16099525	16103169	3645	2922	7	1114	123.46	6.41	1
VrLRR_RLK-38	Vradi07g00001350	16138091	16141487	3397	1527	7	1114	122.33		1
VrLRR_RLK-39	Vradi08g00000540	3725060	3725818	759	549	8	988	109.79	5.79	1
VrLRR_RLK-40	Vradi09g00000367	8973260	8973553	294	294	9	680	76.27	6.54	1
VrLRR_RLK-41	Vradi09g00000606	13594570	13594863	294	294	9	1101	124.18	6.46	1
VrLRR_RLK-42	Vradi09g00000615	13684182	13686370	2189	843	9	1258	141.55	7.53	1
VrLRR_RLK-43	Vradi09g00002473	36363503	36363988	486	486	9	612	68.53	5.55	1
VrLRR_RLK-44	Vradi10g00001185	18348747	18351531	2785	897	10	584	65.74	5.53	1
VrLRR_RLK-45	Vradi11g00000925	8782787	8783011	225	225	11	635	70.12	5.92	1
VrLRR_RLK-46	VradiU00001261	558716	561486	2771	1197	scf	856	93.6	6.96	1

**Table 2:** List of primers used in qRT-PCR analysis.

Primer Name	Forward Sequence (5′-3′)	Reverse Sequence (5′-3′)
VrNBS_NLRRTr-8	AGTGTGGTTGCCGTGTGATA	GGGAGAGGATGTGTTTGGAG
VrNBS_NLRRTir-11	TGGAGTGACCAATCTCAGCA	CCCCTCATCATCTTTTGGAC
VrNBS_NLRRcc-5	ACAGGGACACCCATGACAAT	GGGGTAGAGGACCCATTTGA
VrNBS_TNLRR-8	CCCGAGCTTCCAACAATACA	CATCCGAGGGACCAACTAAA
VrNBS_CNLRR-4	GTCAGGAAAAAGGGGTCTCC	TTTTTGGCTTATTAGACTGACTT
VrLRR_RLK-16	TGG CCC CGG AGG TAT GTG	CCA TCC CCA TGT GTC AGC A
VrLRR_RLK-17	CAA CAA TTC GCT TAG TGG GC	GCT TGG CAT CTG AGA GAG CT
VrLRR_RLK-30	CGA TGG CCG GAA ACG TGT C	GAC AAA CAG GCT AAG CAG GC
VrLRR_RLK-18	GGG TCC ACT GCC CAA CAT TC	GCA TGG AAG TGC CCA AAC C
VrLRR_RLK-20	GTT TCC AAT GCC ACC ACT CTG	GGT GTG AGA GGT GTT GGT CC
VrLRR_RLK-19	GAC GGA GAT GTT CAG GGT G	GCG AGT GAT TCT TCC CTG AAC
VrLRR_RLK-31	GGA GTT GTG TGG GAA GAA AG	GTC ACT CTT TCC CAG GAG C

## Data Availability

All the data have been presented in the manuscript and its Supplementary Files.

## Supplementary Materials

The following supporting information can be downloaded at https://www.mdpi.com/article/10.3390/plants13243601/s1: Table S1: Homology modeling of LRR and LRR-RLK proteins; Table S2: KEGG and STRING analysis of selected LRR and LRR-RLK candidates for protein function.
