# Supplementary material for: Salicylic Acid-Induced Expression Profiles of LRR and LRR-RLK Candidate Genes Modulate Mungbean Yellow Mosaic India Virus Resistance in Blackgram and Its Two Wild Non-Progenitors
Source: Plants (Basel). 2024 Dec 23;13(24):3601. doi: 10.3390/plants13243601 (PMC11678391; doi:10.3390/plants13243601)
Supplement: Supplementary file 1 [file plants-13-03601-s001.zip › plants-3288988-supplementary.pdf]

Table S1: Homology modelling of LRR and LRR-RLK proteins

Sequences	Model	PABS domain-containing protein of	Gene	AlphaFold DB model	Seq Identity	Organism	Oligo-State	GMQE
VrLRR_RLK-16	Model 1	I1MX76.1.A	100815510	I1MX76_SOYBN	93.18%	*Glycine max* (Soybean) (*Glycine hispida*))	Monomer	0.88
	Model 2	6bq2.1.A	Thermospermine Synthase (MtTSPS)	-	91.9%	*Medicago truncatula*	Monomer	0.81
VrLRR_RLK-17	Model 1	K7MQW8.1.A	Uncharacterized protein 100801304	K7MQW8_SOYBN	72.62%	*Glycine max* (Soybean) (*Glycine hispida*)	Monomer	0.60
VrLRR_RLK-18	Model 1	A0A1S3V2X4.1.A	Probable leucine-rich repeat receptor-like protein kinase At1g35710 (LOC106771108), organism:	A0A1S3V2X4_VIGRR	100%	*Vigna radiata* var *radiata* (Mung bean) (*Phaseolus aureus*)	Monomer	0.87
VrLRR_RLK-19	Model 1	I1M1N4.1.A non-specific serine/threonine protein kinase	I1M1N4_SOYBN	100305400,	81.96%	*Glycine max* (Soybean) (*Glycine hispida*)	Monomer	0.84
VrLRR_RLK-30	Model 1	A0A6P4DFC2.1.A Pentatricopeptide repeat-containing protein At5g42450, mitochondrial-like	A0A6P4DFC2_ARADU	OC107491110	75%	*Arachis duranensis* (Wild peanut))	Monomer	0.83
VrLRR_RLK-31	Model 1	A0A1S3VXR8.1.A Receptor-like protein 12 isoform X1	A0A1S3VXR8_VIGRR	LOC106779556,	100	*Vigna radiata* var *radiata* (Mung bean) (*Phaseolus aureus*)	Monomer	0.79
VrNBS_CNLRR-1	Model 1	A0A0R0F2W8.1.A AAA+ ATPase domain-containing protein:	A0A0R0F2W8_SOYBN	-	72.3%	*Glycine max* (Soybean) (*Glycine hispida*))	Monomer	0.69
VrNBS_CNLRR-4	Model 1	I1LWA0.1.A TIR domain-containing protein	I1LWA0_SOYBN	100793798	79.02%	*Glycine max* (Soybean) (*Glycine hispida*)	Monomer	0.79
rNBS_NLRRtir-11	Model 1	I1L618.1.A NB-ARC domain-containing protein	I1L618.1_SOYBN	I1L618.1_SOYBN	82.5%	*Glycine max* (Soybean) (*Glycine hispida*)	Monomer	0.76

Table S2: KEGG and STRING analysis of selected LRR and LRR-RLK candidates for protein function

Protein	Gene	Function	Score
VrLRR_RLK-16	LOC106767222	S-adenosylmethionine decarboxylase proenzyme.	0.837
	LOC106775526	S-adenosylmethionine decarboxylase proenzyme.	0.837
	LOC106759397	S-adenosylmethionine decarboxylase proenzyme.	0.831
	LOC106777763	S-adenosylmethionine decarboxylase proenzyme.	0.831
	LOC106752740	Root meristem growth factor 9.	0.820
	LOC106761805	Ornithine decarboxylase-like; Belongs to the Orn/Lys/Arg decarboxylase class-II family.	0.802
	LOC106764834	Glutamate decarboxylase; Belongs to the group II decarboxylase family.	0.780
	LOC106774034	Arginase 1, mitochondrial; Belongs to the arginase family.	0.743
	LOC106777054	Probable N-acetyl-gamma-glutamyl-phosphate reductase, chloroplastic.	0.682
	LOC106753045	Probable polyamine oxidase 2 isoform X1.	0.680
VrLRR_RLK-17	LOC106773767	Uncharacterized protein LOC106773767.	0.517
	LOC106755526	Pectinesterase inhibitor 9-like.	0.502
	LOC106761005	21 kDa protein-like.	0.490
	LOC106775202	Uncharacterized protein LOC106775202.	0.490
	LOC106760949	Uncharacterized protein.	0.483
	LOC106755267	21 kDa protein.	0.482
	LOC106759705	Uncharacterized protein LOC106759705.	0.478
	LOC106755735	21 kDa protein.	0.474
	LOC106767979	Uncharacterized protein LOC106767979.	0.468
	LOC106780643	Uncharacterized protein LOC106780643.	0.468
VrLRR_RLK-18	LOC106761123	BRI1 kinase inhibitor 1.	0.446
	LOC106764132	BRI1 kinase inhibitor 1-like.	0.446
	LOC106771909	Somatic embryogenesis receptor kinase 2; Belongs to the protein kinase superfamily. Ser/Thr protein kinase family.	0.412
	LOC106772093	Somatic embryogenesis receptor kinase 1 isoform X1; Belongs to the protein kinase superfamily. Ser/Thr protein kinase family.	0.412
VrLRR_RLK-19	LOC106756422	Myb-related protein 305-like.	0.579
	LOC106756984	Transcription factor JAMYB-like.	0.579
	LOC106769219	Vesicle-fusing ATPase.	0.573
	LOC106757626	Calmodulin-3.	0.567
	LOC106758782	Probable calcium-binding protein CML45.	0.567
	LOC106770817	Calmodulin-like protein 11.	0.567
	LOC106775405	Calmodulin-like protein 11.	0.567
	LOC106777484	Probable calcium-binding protein CML45.	0.567

	LOC106757751	Spermatogenesis-associated protein 20 isoform X1	0.547
	LOC106777509	Serine/threonine-protein kinase TOR; Belongs to the PI3/PI4-kinase family.	0.543
VrLRR_RLK-20	LOC106761123	BRI1 kinase inhibitor 1.	0.446
	LOC106764132	BRI1 kinase inhibitor 1-like.	0.446
	LOC106771909	Somatic embryogenesis receptor kinase 2; Belongs to the protein kinase superfamily. Ser/Thr protein kinase family.	0.425
	LOC106772093	Somatic embryogenesis receptor kinase 1 isoform X1; Belongs to the protein kinase superfamily. Ser/Thr protein kinase family.	0.425
VrLRR_RLK-30	LOC106769395	Phosphatidylinositol glycan anchor biosynthesis class U protein.	0.398
	LOC106754218	Pentatricopeptide repeat-containing protein At2g20710, mitochondrial.	0.388
	LOC106757943	Kinesin-like protein KIN-5C; Belongs to the TRAFAC class myosin-kinesin ATPase superfamily. Kinesin family.	0.325
	LOC106778430	Kinesin-like protein KIN-5C.	0.325
	LOC106760114	PH-interacting protein isoform X1.	0.314
	LOC106767863	Late embryogenesis abundant protein At1g64065-like.	0.314
	LOC106772005	Bromodomain and WD repeat-containing protein 3 isoform X1.	0.314
	LOC106775872	Regulatory protein NPR3.	0.312
	LOC106773895	Dormancy-associated protein homolog 4 isoform X1.	0.302
	LOC106762495	Gamma-tubulin complex component; Gamma-tubulin complex is necessary for microtubule nucleation at the centrosome; Belongs to the TUBGCP family.	0.296
VrLRR_RLK-31	LOC106760647	Receptor-like protein 12.	0.479
	LOC106776514	Plant intracellular Ras-group-related LRR protein 3 isoform X1.	0.410
	LOC106770428	TMV resistance protein N-like.	0.405
VrNBS_CNLRR-1	LOC106765277	Sulfate transporter 1.3.	0.237
	LOC106776137	High affinity sulfate transporter 1.	0.237
	LOC106762234	Sulfate transporter 1.3 isoform X1.	0.237
	LOC106775144	High affinity sulfate transporter 2.	0.237
	LOC106772806	RAN GTPase-activating protein 1.	0.216
	LOC106780413	RAN GTPase-activating protein 2.	0.216
	LOC106760912	Serine/threonine-protein kinase Aurora-2; Belongs to the protein kinase superfamily.	0.200
	LOC106766693	Serine/threonine-protein kinase Aurora-3 isoform X1; Belongs to the protein kinase superfamily.	0.200
	LOC106770905	Serine/threonine-protein kinase Aurora-3 isoform X1; Belongs to the protein kinase superfamily.	0.200
	LOC106778956	Serine/threonine-protein kinase Aurora-3 isoform X1; Belongs to the protein kinase superfamily.	0.200
VrNBS_CNLRR-4	LOC106753240	Uncharacterized protein LOC106753240.	0.411
VrNBS_NLRRcc-5	LOC106765277	Sulfate transporter 1.3.	0.237
	LOC106776137	High affinity sulfate transporter 1.	0.237
	LOC106762234	Sulfate transporter 1.3 isoform X1.	0.237
	LOC106775144	High affinity sulfate transporter 2.	0.237

	LOC106772806	RAN GTPase-activating protein 1.	0.216
	LOC106780413	RAN GTPase-activating protein 2.	0.216
	LOC106760912	Serine/threonine-protein kinase Aurora-2; Belongs to the protein kinase superfamily.	0.200
	LOC106766693	Serine/threonine-protein kinase Aurora-3 isoform X1; Belongs to the protein kinase superfamily.	0.200
	LOC106770905	Serine/threonine-protein kinase Aurora-3 isoform X1; Belongs to the protein kinase superfamily.	0.200
	LOC106778956	Serine/threonine-protein kinase Aurora-3 isoform X1; Belongs to the protein kinase superfamily.	0.200
VrNBS_TNLRR-8	LOC106769329	Protein WVD2-like 4 isoform X1.	0.373
	LOC106762703	F-box/LRR-repeat protein At5g63520.	0.368
	LOC106756314	Uncharacterized protein At5g41620.	0.359
	LOC106764163	Uncharacterized protein LOC106764163.	0.359
	LOC106780284	Transcription factor UNE10 isoform X1.	0.355
	LOC106771167	Senescence-associated carboxylesterase 101 isoform X1.	0.346
	LOC106757598	Uncharacterized protein LOC106757598 isoform X1.	0.344
	LOC106778838	Uncharacterized protein LOC106778838.	0.338
	LOC106772454	Putative zinc finger protein At1g68190 isoform X1.	0.334
	LOC106757926	Uncharacterized protein At5g41620.	0.330
VrNBS_NLRRtir-11	LOC106769329	Protein WVD2-like 4 isoform X1.	0.412
